# Endoplasmic reticulum stress in breast cancer: a predictive model for prognosis and therapy selection

**DOI:** 10.3389/fimmu.2024.1332942

**Published:** 2024-02-19

**Authors:** Bin Yang, Shu Wang, Yanfang Yang, Xukui Li, Fuxun Yu, Tao Wang

**Affiliations:** ^1^ Research Laboratory Center, Guizhou Provincial People’s Hospital, Guiyang, Guizhou, China; ^2^ NHC Key Laboratory of Pulmonary Immune-Related Diseases, Guizhou Provincial People’s Hospital, Guizhou University, Guiyang, Guizhou, China; ^3^ Department of Breast Surgery, Guizhou Provincial People’s Hospital, Guiyang, Guizhou, China

**Keywords:** breast cancer, endoplasmic reticulum stress, prognosis, immunotherapy, methotrexate

## Abstract

**Background:**

Breast cancer (BC) is a leading cause of mortality among women, underscoring the urgent need for improved therapeutic predictio. Developing a precise prognostic model is crucial. The role of Endoplasmic Reticulum Stress (ERS) in cancer suggests its potential as a critical factor in BC development and progression, highlighting the importance of precise prognostic models for tailored treatment strategies.

**Methods:**

Through comprehensive analysis of ERS-related gene expression in BC, utilizing both single-cell and bulk sequencing data from varied BC subtypes, we identified eight key ERS-related genes. LASSO regression and machine learning techniques were employed to construct a prognostic model, validated across multiple datasets and compared with existing models for its predictive accuracy.

**Results:**

The developed ERS-model categorizes BC patients into distinct risk groups with significant differences in clinical prognosis, confirmed by robust ROC, DCA, and KM analyses. The model forecasts survival rates with high precision, revealing distinct immune infiltration patterns and treatment responsiveness between risk groups. Notably, we discovered six druggable targets and validated Methotrexate and Gemcitabine as effective agents for high-risk BC treatment, based on their sensitivity profiles and potential for addressing the lack of active targets in BC.

**Conclusion:**

Our study advances BC research by establishing a significant link between ERS and BC prognosis at both the molecular and cellular levels. By stratifying patients into risk-defined groups, we unveil disparities in immune cell infiltration and drug response, guiding personalized treatment. The identification of potential drug targets and therapeutic agents opens new avenues for targeted interventions, promising to enhance outcomes for high-risk BC patients and paving the way for personalized cancer therapy.

## Introduction

Breast cancer (BC) is the most preventable malignancy in women, with high morbidity and mortality (1). At present, the therapeutic approaches have some achievements in BC treatment. The prognosis and therapy of advanced BC patients still have a lot of room for improvement (2). The abundant prognostic models targeted BC have been developed based on distinctive perspectives. For example, Amiri Souri et al. employed machine learning techniques on breast cancer transcriptomics to classify tumors into prognostic categories, showcasing the use of advanced computational methods in prognosis (3). However, the prognosis of BC patients is still unsatisfactory. Hence, it is urgent to explore an effective and accurate prognostic model to enhance the effectiveness of prognosis and aid therapy for BC patients.

Endoplasmic reticulum stress (ERS) is a double-edged sword in cancer development, playing a critical role in both promoting and inhibiting tumor growth. For instance, the study by Nan et al. found that inhibition of ERS in triple-negative breast cancer (TNBC) cells suppressed cell viability, migration, and invasion, indicating a key role of ERS in maintaining the aggressiveness of cancer cells (4). However, Rivera Ruiz et al. demonstrated that inducing ERS in breast cancer cells led to cell cycle arrest and apoptosis, particularly affecting angiogenesis essential for tumor growth and metastasis (5). ERS is also associated with resistance to anti-cancer treatments. It is involved in complex mechanisms that contribute to the survival of cancer cells under therapeutic stress. This aspect is crucial for understanding the role of ERS in tumor progression and treatment response. Recent research elucidates that estrogen modulates the ERS pathway in breast cancer cells, highlighting a novel mechanism by which estrogen influences BC cell survival and proliferation (6). In addition, ERS is also capable of regulating immune cells including macrophages (7), and DCs (8) to affect the development of BC.

The predictive models based on ERS regulators had been widely utilized in some tumors. Fox instance, Zhao et al. established an osteosarcoma prediction model based on six ERS-related genes that is helpful in directing personalized treatment (9). Moreover, Wu et al. improved the prognosis of bladder cancer patients using ERS-related lncRNAs (10). In breast cancer, Fan et al. have reported that ERS prognostic model is associated with the prognosis of BC patients (11). While our model employs advanced statistical and machine learning methods with being further validated in multiple independent cohorts, which may offer more nuanced insights into ERS in breast cancer compared to Fan et al.’s approach. Furthermore, we provide an in-depth analysis of immune cell infiltration disparities and potential drug targets in breast cancer, aspects that are less explored in Fan et al.’s study. Our study also goes further in discussing the practical implications of ERS in breast cancer, particularly in the context of immunotherapy and chemotherapeutic response. Therefore, these points emphasize the unique contributions and potential impact of our research in the field of breast cancer and ERS. Our study’s integration of complex data analysis methods, along with a focus on clinical applicability, marks it as a significant advancement over existing research.

## Materials and methods

### Data collection

To mitigate potential instability arising from batch effects between tumor and normal samples during differential analyses, a careful strategy was implemented. Specifically, gene expression profiles were obtained from the TCGA and GTEx projects, which have undergone recomputation by the UCSC Xena project (12). Notably, this recomputation adhered to a well-defined pipeline, ensuring a robust and standardized approach to data preparation.

We further sourced data from various databases to build and validate our models. The training dataset was compiled from the TCGA database, incorporating gene profiles, mutational landscapes, and clinical details of breast cancer cases. In the preparation of our training dataset, a critical step was the exclusion of samples lacking complete survival information, which is essential for the development of a robust prognostic model. Initially, all collected samples underwent a thorough screening process. We specifically checked for the availability of key survival information, including both overall survival time and status (alive or deceased). Then, samples that were missing either of these critical survival data points were identified and systematically excluded from the dataset.

To augment our findings, we acquired additional validation datasets. These encompassed samples from the Metabric dataset and the GEO databases (GSE202203 and GSE96058). Furthermore, a distinct immunotherapy cohort specifically targeting PD-L1 was obtained from a published study utilizing the IMvigor210CoreBiologies package in R (13).

### Single-cell analysis

Single-cell data on breast cancer was procured from the GEO database under the accession number GSE161529 (14). To establish a robust foundation for analysis, several preprocessing steps were executed. Our initial step involved the elimination of genes that exhibited no expression across all cases (with a count of 0). Subsequently, we applied normalization to the gene expression matrix, employing the “SCTransform” function within the Seurat R package. To gain deeper insights, we conducted Principal Component Analysis (PCA), tSNE and UMAP analysis. To categorize cells, we harnessed the capabilities of the “FindNeighbors” and “FindClusters” functions. To enhance data accuracy, the identification and removal of doublets were undertaken, utilizing the DoubletFinder R package (15).

To further refine the dataset, we excluded cells that exceeded a mitochondrial gene content of 15% or had a gene count below 500. Following these quality control measures, approximately 30 thousand cells remained for subsequent analyses. We employed Celltypist for cell type assignment in our single-cell analysis. Celltypist is a state-of-the-art tool designed for accurate and efficient cell type classification in single-cell RNA sequencing (scRNA-seq) data (16). This comprehensive approach ensured the robust processing and analysis of the single-cell data.

### Functional analysis and pathway exploration

To unravel the intricate landscape of differential expression of ERS regulators between tumors and normal tissues, we utilized the power of the GO and KEGG databases (17, 18). This enabled us to conduct a comprehensive assessment of functional activities and pathways. Leveraging the capabilities of the Enrichplot package within the R environment, we embarked on this pivotal analysis. Enrichplot is an R package designed to provide a rich and intuitive visual representation of complex biological data, particularly useful in the context of gene-set enrichment analysis.

In addition, the clusterProfiler algorithm was instrumental in facilitating the execution of Gene Set Enrichment Analysis (GSEA) between the distinct risk subgroups (19). This dynamic approach illuminated the underlying functions differentiating the two subgroups. To establish statistical significance, we deemed a False Discovery Rate (FDR) value below 0.05 as indicative of noteworthy findings. It is noteworthy that our robust methodology involved the performance of 1,000 permutations to enhance the robustness of our results.

### Establishment of the ERS score

To unravel the implications of ERS in BC, a systematic approach was adopted. We initiated this exploration by conducting a differential analysis, specifically comparing gene expression patterns between tumor and normal tissues within the GTEx-TCGA dataset.

To visually represent the differential gene expression outcomes, a heatmap was employed, effectively illustrating the disparities. Concurrently, an analysis of gene correlations was carried out, facilitated by the utilization of the igraph package. The pivotal ERS score was then meticulously computed. This calculation was anchored in the differentially expressed ERS regulators. In this endeavor, the ssGSEA algorithm was employed for bulk data (20), while the Ucell algorithm was harnessed for single-cell data (21). This dual-pronged approach ensured a comprehensive and robust assessment of the ERS score, facilitating a deeper understanding of its role within the realm of BC, considering both the collective behavior of tumor cells (bulk analysis) and the heterogeneity at the single-cell level.

### Development and validation of ERS-model

The evolution and validation of the ERS-model underwent a meticulous process to ascertain its predictive efficacy in the context of BC.

To identify potent ERS regulators with predictive capabilities, we initiated a univariate Cox regression analysis on differentially expressed ERS regulators. This analysis took place within a dedicated training set, culminating in the selection of eight ERS regulators intimately associated with BC outcomes. A robust evaluation of BC prognosis was facilitated through the assessment and computation of Overall Survival (OS) in BC patients.

For constructing the ERS-model, a lasso regression approach was meticulously deployed. This framework adeptly extracted essential ERS regulators. These elements were thoughtfully combined to forge an ERS-model, a pivotal tool for gauging BC patient outcomes. The resultant risk scores were determined through a mathematical formulation:


$$riskcore\;=\;\sum_{i\;=\;1}^{n}\left(\beta_{i}\;\times \;Exp_{i}\right)$$

wherein ‘n’ represents the number of ERS regulators, ‘Exp’ signifies the ERS gene profile, and ‘β’ denotes the multi-Cox coefficient. After this, patients were categorically classified into distinct risk subgroups based on their corresponding risk scores.

To robustly gauge the generality of the risk profile, external datasets were harnessed. These datasets effectively served as validation sets, further strengthening the model’s credibility. Employing R v4.2 and adopting Kaplan-Meier (KM) survival analysis, we meticulously examined the discernible variation in outcomes between the identified risk subgroups. The statistical significance of this variation was established through a p-value criterion (P < 0.05), lending depth and validity to the prognostic capacity of the developed ERS-model.

### Genomic alteration landscape analysis

To unravel the genomic alteration disparities between the ERS-model subgroups, an extensive examination of mutation and Copy Number Alteration (CNA) data was conducted within the TCGA-BRCA dataset. We began by extracting the raw mutation file and proceeded to calculate the Tumor Mutation Burden (TMB) for each sample. To offer insights into the genetic landscape, the top 28 genes (mutational rate > 5%) were visually represented utilizing the maftools package. Following the methodology described by Wang et al. (22), we employed the deconstructSigs package to derive mutational signatures unique to each patient. We then highlighted four with notable occurrence frequencies in BRCA: SBS1, SBS3, SBS11 and SBS12. By including these specific mutational signatures, our study aims to comprehensively analyze the mutational processes that are most relevant to ERS in breast cancer. This approach not only enhances the depth of our genomic analysis but also provides insights into potential therapeutic targets and prognostic markers associated with ERS in breast cancer. Moreover, the top 5 regions exhibiting a broad-level CNA frequency were meticulously selected. Particularly, the focus was placed on genes within chromosomes 9p21.3 (CDKN2A, CDKN2B, MTAP and IFNA1).

### Analyses of TME variations

Six algorithms, including MCPcounter (23), EPIC (24), xCell (25), CIBERSORT (26), quanTIseq (27) and TIMER (28), were employed to quantify the abundance of distinct immune cell types using the IOBR package (29). Additionally, ESTIMATE and TIDE were utilized to assess the tumor microenvironment’s composition, structure, and state, providing crucial insights into the tumor’s biological traits and prognosis (30, 31). Lastly, the expression levels of multiple immunoregulatory genes were examined to discern variations in immune competence.

### Estimation of drug targets and chemotherapeutic response

We obtained comprehensive target data for 6,125 compounds from the Drug Repurposing Hub (https://clue.io/repurposing), resulting in 2,249 distinct drug targets after removing duplicates (32). Spearman correlation analysis pinpointed potential drug targets linked to unfavorable prognosis by correlating gene expression of targetable genes with risk scores (correlation coefficient > 0.25, P < 0.05). A positive correlation indicates that as the expression of these genes increases, so does the risk score, suggesting a potential role in driving unfavorable prognosis. Subsequently, CERES scores were correlated with risk scores for brain cell lines from CCLE, identifying genes (correlation coefficient < -0.2, P < 0.05) associated with poor prognosis dependence (33). This additional analysis further refined our identification of potential drug targets, focusing on those genes most relevant to adverse outcomes in breast cancer.

Leveraging CTRP and PRISM datasets, containing extensive drug screening and molecular data across cancer cell lines, enabled precise drug response prediction. Differential expression analyses were conducted between bulk and cell line samples. For drug response prediction, the reliable ridge regression model within the pRRophetic package was used. Trained on expression profiles and drug response data from solid Cancer Cell Lines (CCLs), this model exhibited robust performance validated by default 10-fold cross-validation (34).

Connectivity Map (CMap) analysis gauged the therapeutic potential of candidate agents in BC (35). Differential gene expression analysis between tumor and normal tissue samples was followed by submitting the top 300 genes (150 up-regulated and 150 down-regulated) to the dedicated CMap website (https://clue.io/query), drawing on gene expression signatures from CMap v1 and LINCS database. Negative connectivity scores indicated the potential therapeutic efficacy of the perturbation in the disease context, and suggest that certain compounds could reverse the disease-specific gene expression patterns found in BC, highlighting their therapeutic potential.

### Human sample collections and IHC staining

This study utilized human specimens from a cohort of 30 patients diagnosed with BC. The specimens were collected during surgical procedures at Guizhou Provincial People’s Hospital. HE staining was performed on the collected materials following established protocols. The diagnostic evaluations were carried out independently by two pathologists. Detailed information of cohorts was summarized in **Supplementary Table S1**.

We performed IHC in paraffin-embedded samples according to our previous procedures (36, 37). Antibodies used in this study were listed in **Supplementary Table S2**. The assessment followed established protocols and scoring criteria, with two pathologists independently evaluating protein expression levels, as described in our previous publication (37).

For Hematoxylin and Eosin staining, tissue sections were first deparaffinized and rehydrated. Hematoxylin was applied to stain cell nuclei, followed by a brief wash and application of Eosin, which stains cytoplasmic components. The slides were then dehydrated and mounted for microscopic examination. Immunohistochemistry involved deparaffinizing tissue sections and applying primary antibodies specific to our proteins of interest. After washing, secondary antibodies conjugated with a chromogen were added. The presence of the target protein was visualized as a colored precipitate under a microscope.

### qRT-PCR and patient stratification

Total RNA was extracted from breast cancer tissue samples employing TRIzol reagent (Invitrogen, Carlsbad, CA, USA). Subsequent cDNA synthesis and qRT-PCR utilized GoScript reverse transcriptase and Master Mix (both from Promega), as per manufacturer’s protocols. The CFX96 Touch real-time PCR detection system (BioRad, Hercules, CA, USA) facilitated data acquisition. Quantitative analysis was conducted using the 2^-ΔΔCq^ method, with GAPDH serving as the normalization reference gene.

Based on the expression levels obtained, patients were categorized into low-risk and high-risk groups using a predefined threshold determined by the ERS-model’s formula.

## Results

### Evaluation of ERS-associated genes in BC patients

**Figure 1:**
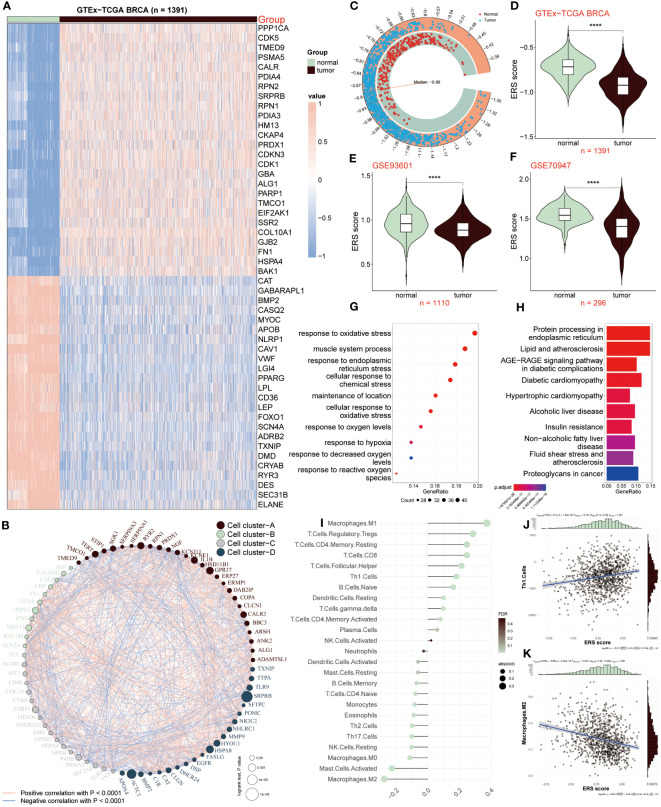
ERS regulators in tumor and non-tumor samples. **(A)** Heatmap showing expression patterns of the top 50 ERS regulators in tumor and normal samples. **(B)** Network depicting interactions among 50 differentially expressed genes related to ERS, categorized into four cell clusters **(A–D)**. Each cluster is color-coded. Circle size reflects the significance of a single regulator’s impact on BC. Red lines indicate positive correlations, while blue lines indicate negative correlations. **(C, D)** Comparison of ERS scores between normal and tumor samples in GTEx-TCGA BRCA dataset. **(E, F)** Comparison of ERS scores in GSE93601 and GSE70947 validation datasets. **(G, H)** GO and KEGG enrichment analysis of distinct ERS-related genes. **(I)** Correlation between infiltrated immune cells and ERS score. **(J)** Correlation of ERS score with Th1 cells. **(K)** Correlation of ERS score with M2 macrophages. ****P < 0.0001.

In our current study, we joined TCGA and GTEx databases to screen out differentially expressed genes between the normal and tumor samples, and got 243 differential expressed genes (DEGs) (**Supplementary Table S3**). Among the 50 most notable DEGs related to ERS displayed in the heatmap (**Figure 1A**), we observed that 26 ERS regulators were dramatically up-regulated in tumor samples, with PPP1CA, CDK5, and TMED9 showing fold changes of 1.25, 1.67, and 1.05, respectively. Conversely, the expression levels of the remaining 24 regulators, including CAT, GABARAPL1, and BMP2, were significantly increased in normal groups, exhibiting fold changes of -1.55, -2.03, and -3.05, respectively. To clarify the connection deeply and comprehensively between these distinctive ERS regulators, we clarified 50 regulators into four cell clusters (marked as A, B, C, D), and a well-connected network was constructed representing the relationship among them. Thus, based on this network, an intricate connection among these ERS regulators was explored clearly, of which GFAP and RNF186 obtained from cluster B exhibited a strong and closely positive correlation (cor = 0.56), which meant that both had a synergistic role. In contrast, there was a notably negative correlation between KCNJ11 and RPN1 from cluster A revealing the antagonistic effect (cor = -0.37). In addition, synergism and antagonism also were detected from different clusters. For example, IFNG and FASLG displayed a positive relation (cor = 0.84), while a markedly negative relevance between POMC and HSPA4 was found (cor = -0.43) (**Figure 1B**).

We further calculated the ERS score to illustrate the relationship between ERS and BC, of which the score of the tumor population was below the median (-0.89), whereas it was above the median score in the normal population (**Figure 1C**). The connection between ERS score and distinctive populations was exhibited according to the results from the GTEx-TCGA BRCA dataset and the other two validation datasets (GSE93601 and GSE70947). The result showed that the normal group had a high ERS score, conversely, ERS score was lower in TCGA-BRCA patients relative to healthy samples (**Figure 1D**), which was consistent with the consequences based on GSE93601 and GSE70947 validation datasets (**Figures 1E, F**). Subsequently, we continued to elucidate the functions and pathways of these different regulators associated with ERS between BC patients and normal groups. The GO result revealed that these genes exhibited a close connection with these roles, such as response to oxidative stress, response to endoplasmic reticulum stress, muscle system process, cellular response to chemical stress, oxidative stress et al. (**Figure 1G**). According to the KEGG result, it was concluded that these distinctive genes were mainly abundant in protein processing in endoplasmic reticulum, lipid and atherosclerosis, diabetic cardiomyopathy and other signaling pathways (**Figure 1H**). Through the above results, it was demonstrated these ERS-related genes presented a close connection with the response stress, and the heterogeneity of their expression revealed that ERS regulators may serve as underlying targets in the development and progress of BC.

Since tumor microenvironment (TME) is involved in tumor development, we investigated the relevance between the ERS score and infiltrated immune cells as presented in **Figure 1I**, of which M1 macrophages, Treg and CD8 T cells were positively correlated with ERS score. Oppositely, M2 macrophages, activated mast cells and NK cells appeared a remarkably negative association with ERS score. In addition, the correlation analyses of ESR score with Th1 cells and M2 macrophages were respectively shown (**Figures 1J, K**). Above all, these results announced that BC patients with lower ERS scores, possessed less proportion of immune-infiltrating cells, indicating that this group may be in the immunosuppressive state.

### Decipherment of ERS activity using single-cell

**Figure 2:**
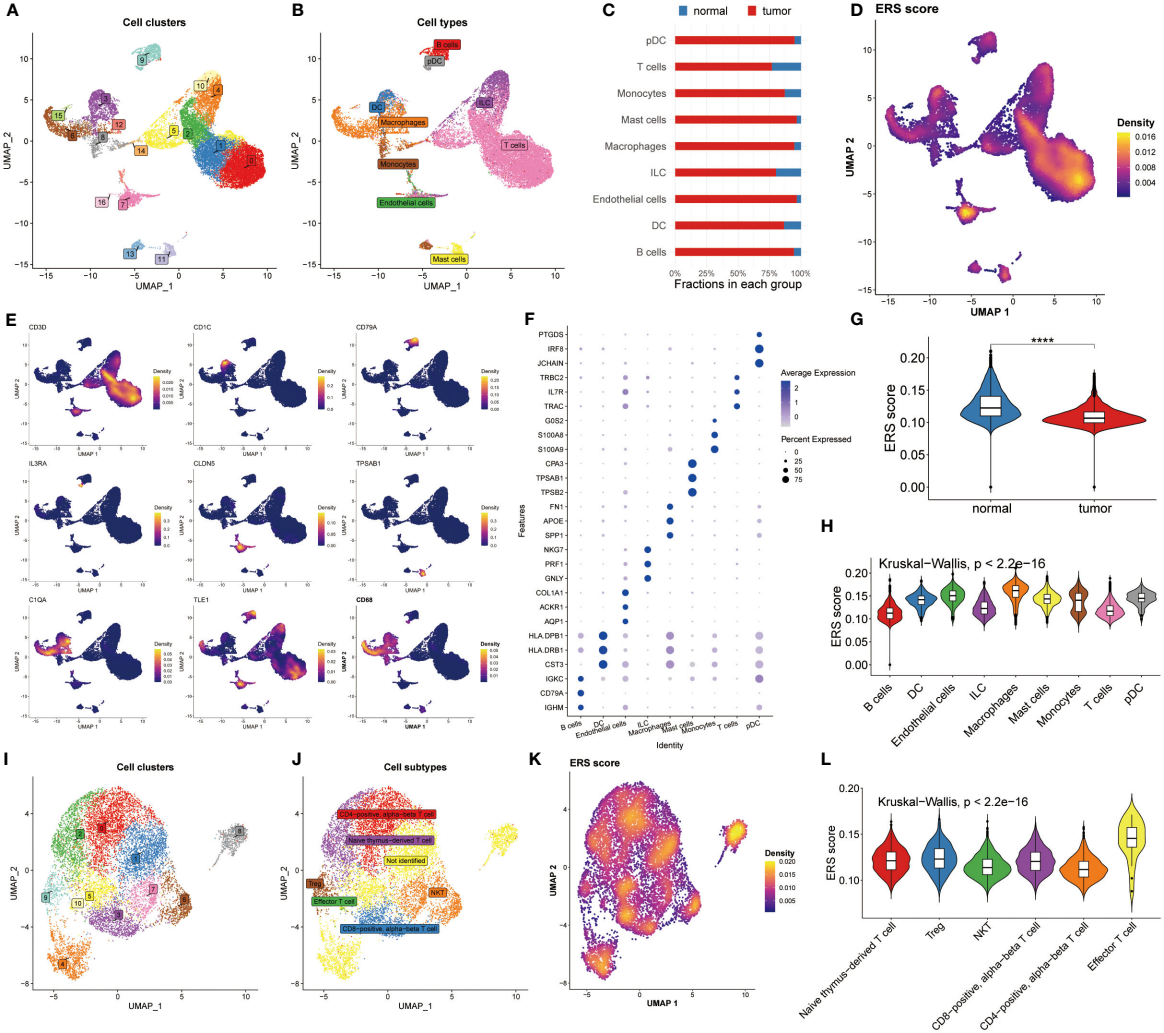
Analysis of ERS in the level of single–cell. **(A)** Distribution of 17 cell clusters derived from normal groups and BC patients. **(B)** Identification of nine distinct cell types using the Celltypist algorithm. **(C)** Bar chart displaying the proportion of the 9 cell types in each group. **(D)** Bar chart displaying the proportion of the 9 cell types in each group. **(E)** Expression features of marker genes for the 9 identified cell types. **(F)** Top 3 differentially expressed markers in each cell type. **(G)** Violin plots highlight the difference in ERS scores between normal and tumor samples. **(H)** Distribution of ERS scores among the nine cell types. **(I, J)** UMAP plots visualizing cell clusters and subtypes within T cell subsets. **(K)** Diversity of ERS scores across different T cell subpopulations. **(L)** Violin plots depicting ERS scores for 6 T cell subsets.

Next, we revealed the expression feature of ERS in diverse immune-infiltrating cells at the level of single-cell. 22195 cells obtained from the normal and BC samples were used, and then seventeen cell clusters were gained as plotted in **Figure 2A**. We then grouped them into nine cell types using the celltypist algorithm (**Figure 2B**). Additionally, we compared the fraction of nine cells selected from normal and BC samples. Interestingly, pDC, macrophages and endothelial cells were abundant in tumor samples rather than in normal groups (**Figure 2C**). This observation highlights the heterogeneity of the tumor microenvironment and underscores the roles of specific immune and stromal cells in BC pathology. These cells were annotated by their maker, for instance, CD3D was utilized to recognize T cells, CD1C was the marker molecule of pDC cells, CD97A was specifically expressed by B cells, CD68 was used to distinguish macrophages, IL3RA was defined by DC, besides, endothelial cell, mast cells, monocytes and ILC were marked by CLDN5, TPSAB1, C1QA, and TLE1, respectively (**Figure 2E**). Moreover, the top differential expressed genes in nine cells were displayed in **Figure 2F**.

The ERS score was then calculated among the nine cell types using the Ucell algorithm (**Figure 2D**). We showed that the normal groups had a higher ERS score compared with BC patients, which was following the bulk results (**Figure 2G**). Later, the interconnection between ERS score and nine cell types was estimated and exhibited in **Figure 2H** based on the Kruskal-Wallis method, suggesting the ERS score presented a significant association with these cells. Because of the specificity of T cell count, we determined to deeply analyze ERS score of T cell subtypes. Accordingly, 11 cell clusters were recognized (**Figure 2I**) and 6 cell subtypes were identified (**Figure 2J**). We recomputed the ERS score based on T cell subsets, manifesting that ERS score significantly distinguished all these subsets (**Figure 2K**). Similarly, The Kruskal-Wallis results of the ERS score in T cell subpopulations proclaimed that the ERS scores were highly enriched in the effector T cell subsets (**Figure 2L**). Conjoint analysis with **Figure 2G** findings, it was found that T cell subtypes were significantly abundant in the normal population, which may be an explanation for the immune suppression status of BC patients. Notably, a detailed analysis of T cell subtypes revealed that effector T cell subsets were enriched in higher ERS scores, suggesting their potential role in counteracting immune suppression observed in BC patients.

### Communications among distinctive cells in the development of BC

**Figure 3:**
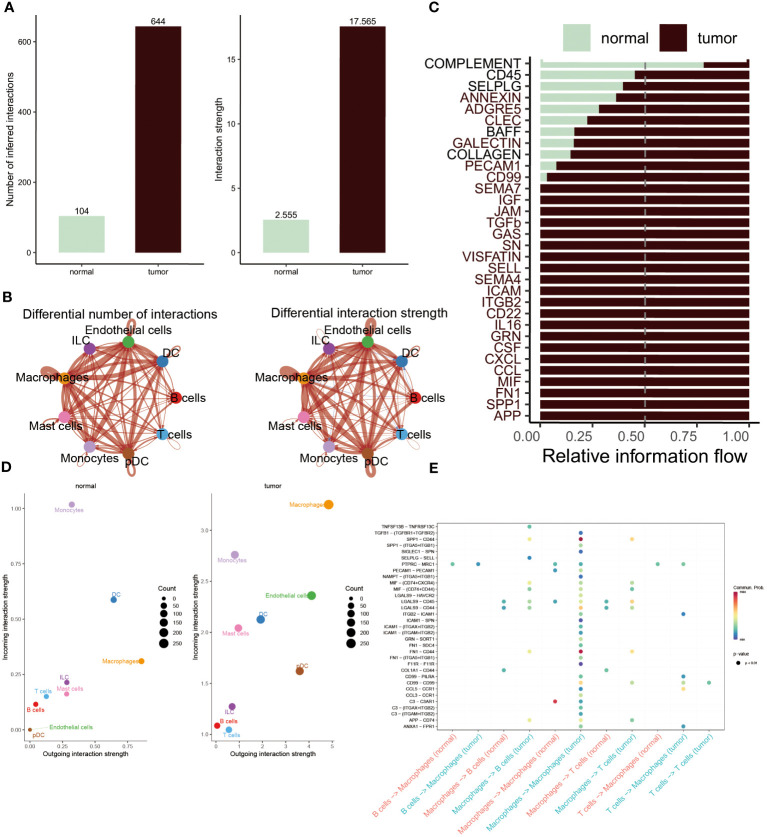
Cell-cell interactions between the normal and BC. **(A, B)** Interaction numbers (left) and interaction strengths (right) of different cell types are displayed through bar charts and circle charts in normal and BC populations. Thicker lines represent stronger relationships, with red and blue colors indicating positive and negative interactions, respectively, in BC patients compared to normal populations. **(C)** Stacked plots showcasing the distribution of signaling pathways in different cells within the two groups. **(D)** Scatter plot illustrating the difference in incoming and outgoing interaction strengths in normal groups (left) and tumor patients (right). Larger circles indicate stronger strengths. **(E)** Dot plot presenting the distribution of distinctive signaling molecules in T cells, B cells, and macrophages between the two groups.

The CellChat analysis was performed to reveal the cell-cell relationships within the progression of BC. According to this result, it was found that the interaction numbers and strength were more prominent in BC patients (**Figure 3A**). More interestingly, except for B and T cells, other cells showed stronger interaction of numbers and strengths in tumor patients relative to normal populations. In contrast, B cells and T cells existed frequent communication with DC cells and ILC cells in healthy groups (**Figure 3B**). Subsequently, the difference in the interaction of different pathways was compared within these two groups. The extraordinary thing was that COMPLEMENT was the only pathway activated in normal populations, whereas other pathways were remarkably activated in BC patients, including CD45, CD99, CCL, IL16 and APP (**Figure 3C**). Furthermore, to constantly detect the changes in submitting or gaining signals between distinctive groups, a comparison based on outgoing and incoming interaction strength in 2D space was conducted. The scatter plot showed that monocytes, DC cells, ILC cells, T cells and B cells served as main sources in normal groups, while macrophages, endothelial cells, mast cells and pDC cells were significant sources in BC patients (**Figure 3D**). Ultimately, the dot plot exhibited stronger interaction possibilities among T cells, B cells and macrophages in BC groups. As the result revealed that almost all molecules were distributed in samples of tumor macrophages, suggesting the interaction of macrophages was the most prominent in tumor samples (**Figure 3E**).

### Construction of ERS-model based on ERS regulators in BC

According to the above results, differently expressed regulators associated with ERS had been obtained. At the same time, the tight connection between the ERS score and infiltrated immune cells and T cells subpopulations in tumor and non-tumor groups were systematically analyzed. It demonstrated that ERS-related genes did exist significant and undeniable relationships with BC, which needed to be further studied and explored. Consequently, we constructed a predictive model that utilized eight ERS-associated genes to assess the prognosis of BC patients.

**Figure 4:**
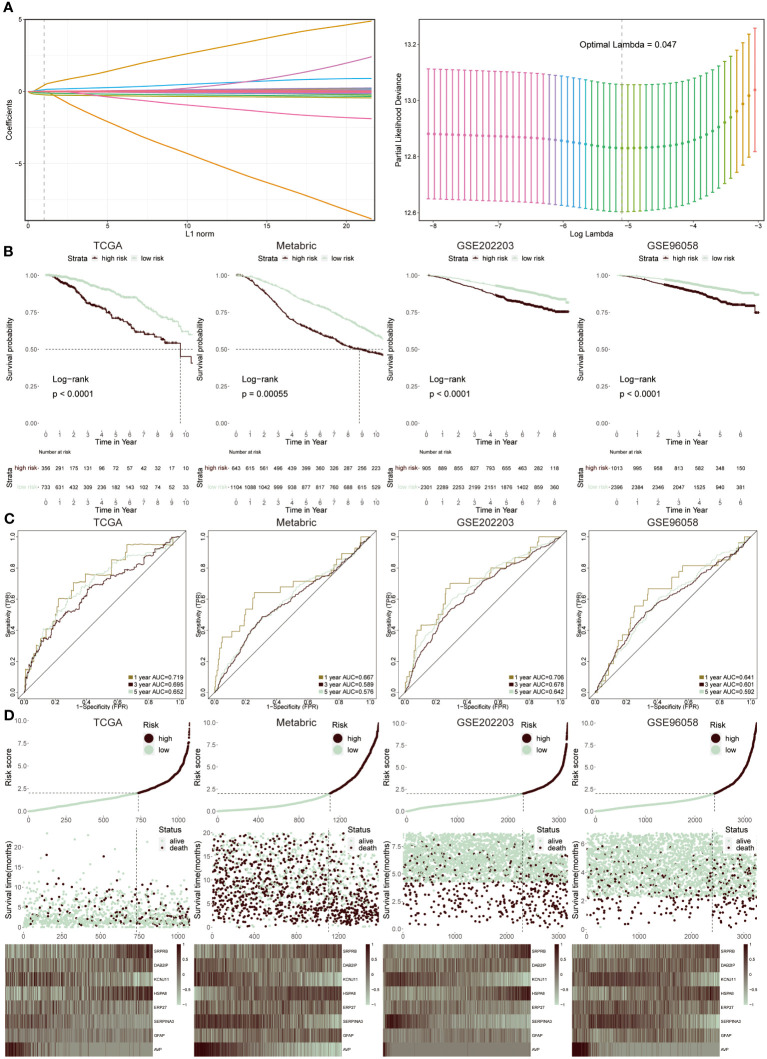
Construction and validation of ERS-model. **(A)** LASSO regression analysis was utilized to screen ERS-associated genes. **(B)** KM survival curves presented the difference in the survival probability between two groups in TCGA, Metabric, GSE202203 and GSE96058 datasets, respectively. **(C)** The ROC curves separately displayed the AUC values at one-, three-, and five-year in four cohorts. **(D)** The correlation between risk score and survival status was clarified in four cohorts. Moreover, the heatmap displayed the expression feature of eight ERS-related genes in each cohort.


We utilized the LASSO regression analysis to screen out 54 ERS regulators with significant prognosis (*P* < 0.05), and the optimal lambda was 0.047 (**Figure 4A**). The integration analysis of the TCGA training cohort and three testing cohorts (Metabric, GSE202203 and GSE96058) were used to construct the ERS-model as the formula exhibited below:

$$\begin{array}{c} risk\;core\;=SRPRB^{*}0.323+DAB2IP^{*}0.196-KCNJ11^{*}0.289+HSPA8^{*}0.339-ERP27^{*}\\ 0.134-SERPINA2^{*}0.665\;+GFAP^{*}0.222-AVP^{*}1.044 \end{array}$$

The selection was based on a comprehensive analysis combining differential gene expression, survival analysis, and literature review to identify genes significantly associated with ERS and BC prognosis. These genes were chosen due to their proven involvement in ERS pathways and their statistically significant correlation with survival outcomes in BC patients. Therefore, two groups were successfully divided via this risk score. Then, to prove the reliability of this ERS-model, the KM survival curve was performed. In the TCGA cohort, the low-risk groups had a better outcome relative to the high-risk groups, which was in accord with the results from the three testing cohorts (**Figure 4B**). Accordingly, the ROC curve was plotted to evaluate the prognostic efficiency of this model for BC patients (**Figure 4C**). The results revealed that in TCGA and GSE202203 cohorts, the range of AUC values was between 0.64 and 0.72, as well as in the other two cohorts, the lowest AUC values were also greater than 0.5, suggesting the predictive ability of ERS prognostic model was efficient and reliable. According to the results from the relevance between risk score and survival status in four datasets, it concluded that the low-risk population possessed a higher likelihood of survival, in contrast, the high-risk group presented increasing populations with dead status (**Figure 4D**). Finally, the heatmap displayed the expression profiles of eight ERS-related genes within two groups, of which SRPR8, DAB2IP, HSPA8 and GFAP exhibited a positive correlation with a risk score, while the remaining genes were dramatically enriched in a low-risk group.

### Assessment of the independence of ERS-model

**Figure 5:**
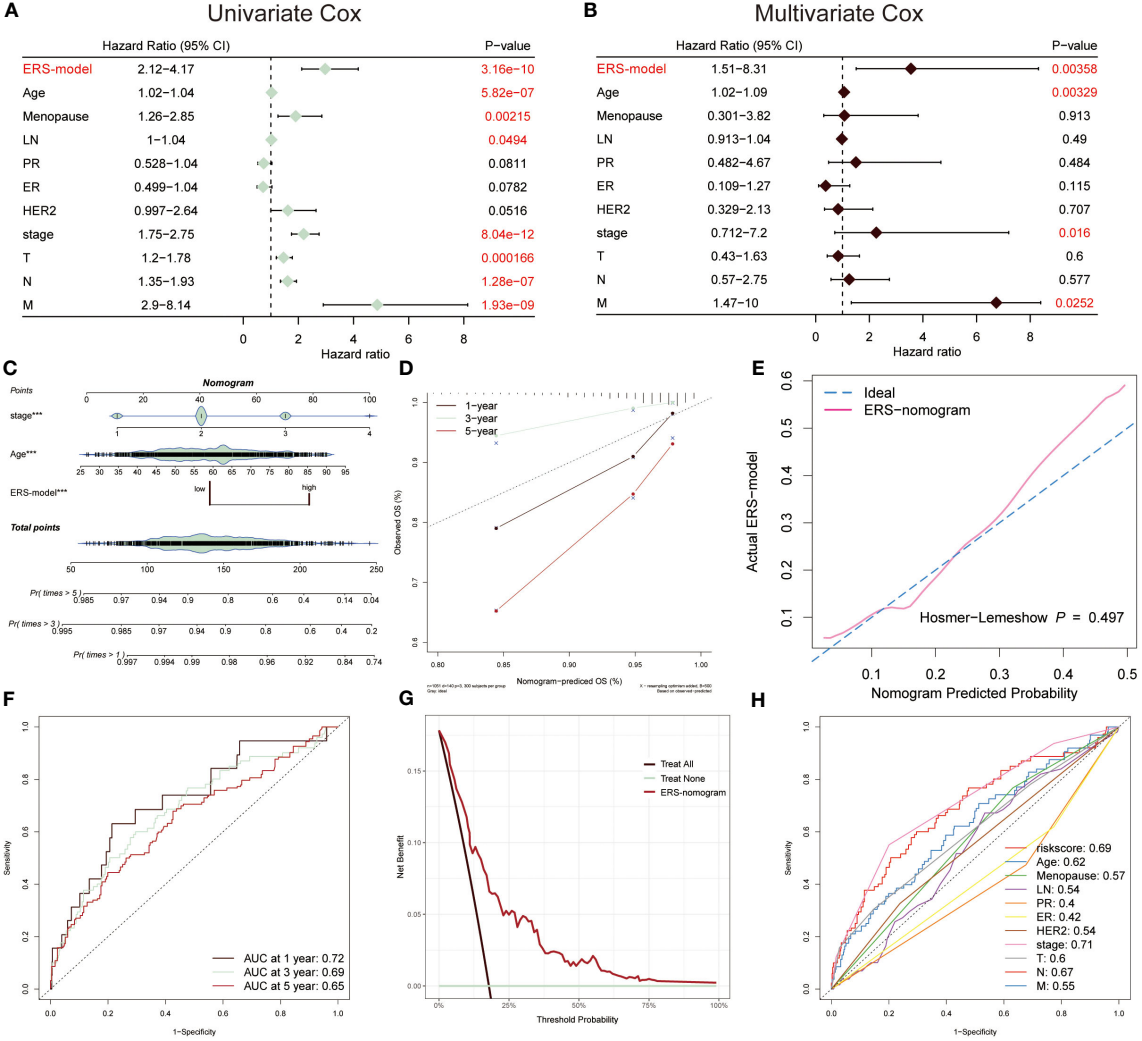
Prognostic characters of ERS-model. The univariate **(A)** and multivariate Cox **(B)** were performed, which consisted of ERS-model, age menopause, LN, PR, ER, HER2, stage, T, N and M. **(C)** An ERS-nomogram was built consisting of risk score, age and stage index. **(D)** The correction curves were plotted based on the observed OS (%) and nomogram-predicted OS (%). **(E)** The Hosmer-Lemeshow was used to estimate the accuracy of the ERS-nomogram in comparison with the ideal curve. **(F)** The AUC values of this nomogram at 1-, 3- and 5-year were 0.72, 0.69 and 0.65, respectively. **(G)** Decision Curve Analysis (DCA) was described, of which the curves were considered as two extreme lines drawn from treat all and treat none, respectively. **(H)** The AUC values from risk scores and other clinical indicators were exhibited by ROC curves.

The univariate and multivariate Cox regression analysis was performed, suggesting that ERS-model, age and stage were able to be separately considered as an independent prognostic index in BC (**Figures 5A, B**). Based on the greater performance and prognostic potential of the ERS-model, a nomogram consisting of risk score and clinical factors, including age and stage, was utilized to predict the survival possibility of BC patients (**Figure 5C**). The results from the correction curve demonstrated that our nomogram was equipped with high accuracy (**Figure 5D**). Furthermore, consistent with the role of **Figure 5D**, the Hosmer-Lemeshow test again testified to the superior accuracy of this nomogram, since there was no statistically remarkable difference between the predicted values of the ERS-nomogram and the ideal observed values (*P* > 0.05), with a very high degree of fit (**Figure 5E**). The ranges of the AUC value based on the ERS-nomogram were between 0.65 and 0.72 (**Figure 5F**), representing that ERS-nomogram possessed a favorable predictive capability. Additionally, the DCA result manifested that the ERS-nomogram curve was above the other two extreme curves (**Figure 5G**). At last, the ROC curves of risk score, age, stage, PR and other clinical factors were described, finding that the AUC value of risk score was relatively higher than other factors, except for stage (**Figure 5H**). Based on the above-mentioned results, demonstrated that ERS-nomogram was equipped with a satisfying predictive performance in BC.

### Comparison of ERS-model with five existing models

**Figure 6:**
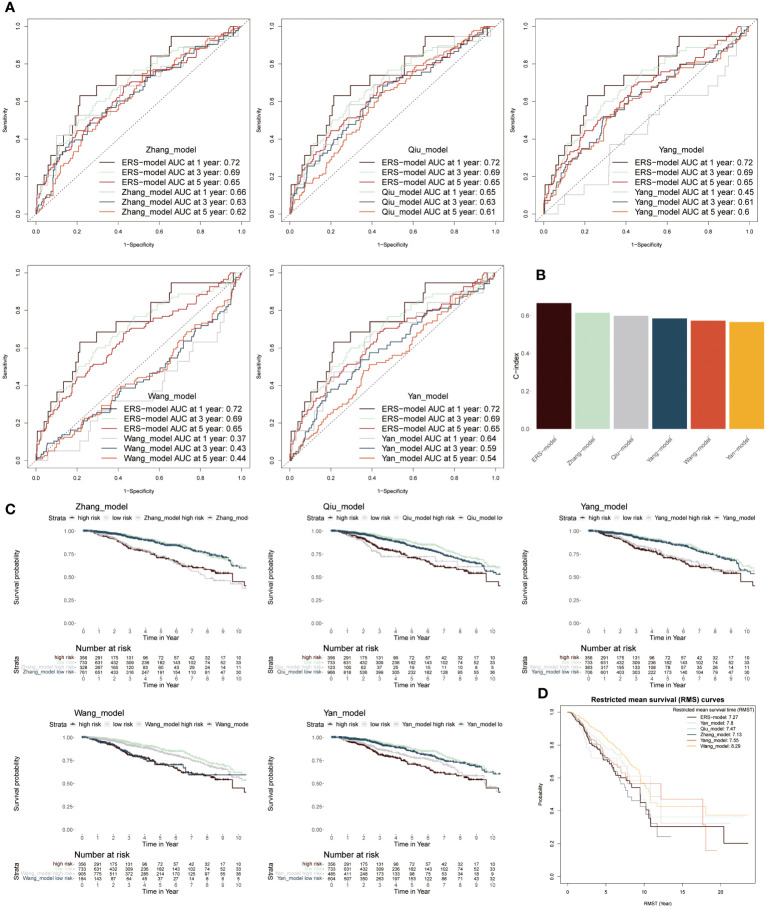
Comparison between ERS-model and five existing models. **(A)** The AUC values from six models in the first-, third- and fifth years were individually described based on the ROC curves. **(B)** The C-index values of six models were singly shown by six colored bar chats. **(C)** To separately compare the KM survival curve of ERS-model with the other five models. **(D)** The RMS curves solely exhibited the RMST values based on six models.

To further stick out the advantages of ERS-model, we selected five well-established models to make a comparison with our ERS-model based on AUC values, KM survival curves and C-index (38–42). Firstly, according to AUC values, the range of AUC values was between 0.62 and 0.66 in the Zhang model. In the Qiu model, it was found that the largest AUC value was still below the AUC value of the ERS-model. The largest AUC values in model Yang, Wang and Yan were 0.61, 0.44 and 0.59, respectively (**Figure 6A**). Based on these results, it preliminary illustrated that ERS-model was superior in prognostic potential. Besides, ERS-model possessed the highest C-index value as compared with the other five models (**Figure 6B**). The KM survival curves displayed that in five models, the survival probability of the low-risk group was highest, at the same time, the high-risk group had the poorest survival advantage (**Figure 6C**). Restricted mean survival time (RMST) is a reasonable and effective appraising method for long-term benefits. The results depicted that ERS model had a longer curve duration and a significant tail elevation, suggesting that it was effective for observing cancer prognosis (**Figure 6D**). In general, the prognostic ability of the ERS-model outperformed other existing models for BC patients.

### Multi-omics analysis of genetic variations based on ERS-model

**Figure 7:**
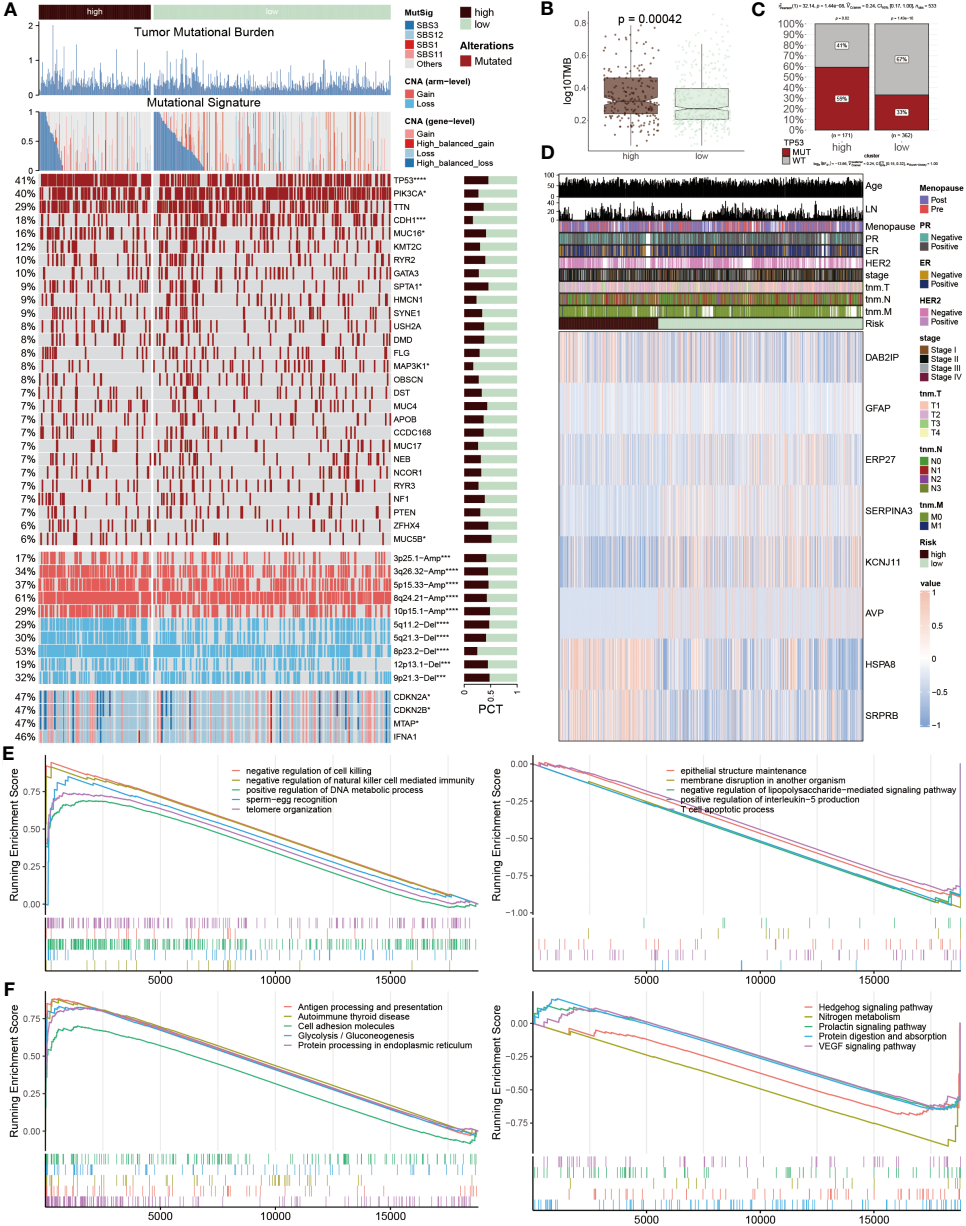
Genetic variations based on ERS-model. **(A)** Differences between the two groups are illustrated, including tumor mutational burden, mutational signature, 28 mutated genes, and the distribution of screened genes within Chr9p21.3. The right bar charts display their proportions. **(B)** TMB values are presented in logarithmic form between the two groups. **(C)** Proportions of TP53 MUT and WT are shown in the two populations, where in the high-risk group, MUT and WT account for 59% and 41% respectively, while in the other population, MUT and WT account for 33% and 67%. **(D)** A heatmap displays the distribution of eight ERS regulators and ten clinicopathological factors in the two populations. **(E, F)** GSEA enrichment results. The GO **(E)** and KEGG **(F)** enrichment outcomes from the high-risk group are displayed on the left, while the corresponding results from the low-risk group are shown on the right.

To systematically assess the genomic heterogeneity based on ERS-model, we calculated the gene mutations and copy number alterations (CNAs) between the ERS subgroup (**Figure 7A**). We observed a dramatically higher tumor mutational burden (TMB) in the high-risk group compared to the lower ones (**Figure 7B**), indicating a greater genomic instability which is often correlated with aggressive tumor behavior and potentially a poorer prognosis. Moreover, the mutation frequency of TP53, PIK3CA, CDH1, MUC16, SPTA1, MAP3K1 and MUC5B were significantly variated between the ERS subgroups (*P* < 0.05) (**Figures 7A, C**). Further analyses showed that more amplifications or deletions were also detected in the high-risk BC patients, for example, the amplification of 3p25.1, 3q26.32, 5p15.33, 8q24.21, and 10p15.1 and the deletion of 5q11.2, 5q21.3, 8p23.2, 12p13.1 and 9p21.3. Moreover, this finding was also confirmed, according to the deletion of the two tumor suppressor genes CDKN2A and CDKN2B within 9p21.3. These genetic alterations provide a clearer picture of the molecular landscape distinguishing ERS subgroups and suggest targets for potential therapeutic intervention.

After that, the heatmap visualized the expression profiles of eight ERS regulators across two groups, of which DAB2IP, GFAP, HSPA8 and SRPR8 exhibited a higher expression level in high-risk patients, distinctively, ERP27, SERPIN3, KCNJ11 and AVP mainly focused on expression in another group (**Figure 7D**), which points to their possible roles in mediating BC’s adverse outcomes through ERS pathways. Subsequently, the function annotation and pathway enrichment were conducted to explore the underlying mechanism of BC development. In high-risk groups, positive regulation of DNA metabolic process, negative regulation of natural killer cell-mediated immunity, negative regulation of cell killing, antigen processing and presentation, cell adhesion molecules and protein processing in endoplasmic reticulum were activated, however, epithelial structure maintenance, negative regulation of lipopolysaccharide-mediated signaling pathway, positive regulation of interleukin-5 production, T cell apoptotic process, Hedgehog signaling pathway and VEGF signaling pathway were inhibited (**Figures 7E, F**). These findings highlight the complex interplay of genetic and immunological factors in BC progression and offer insights into the molecular mechanisms driving disease advancement in high-risk patient.

### Distinctive immune features between the ERS subgroups

**Figure 8:**
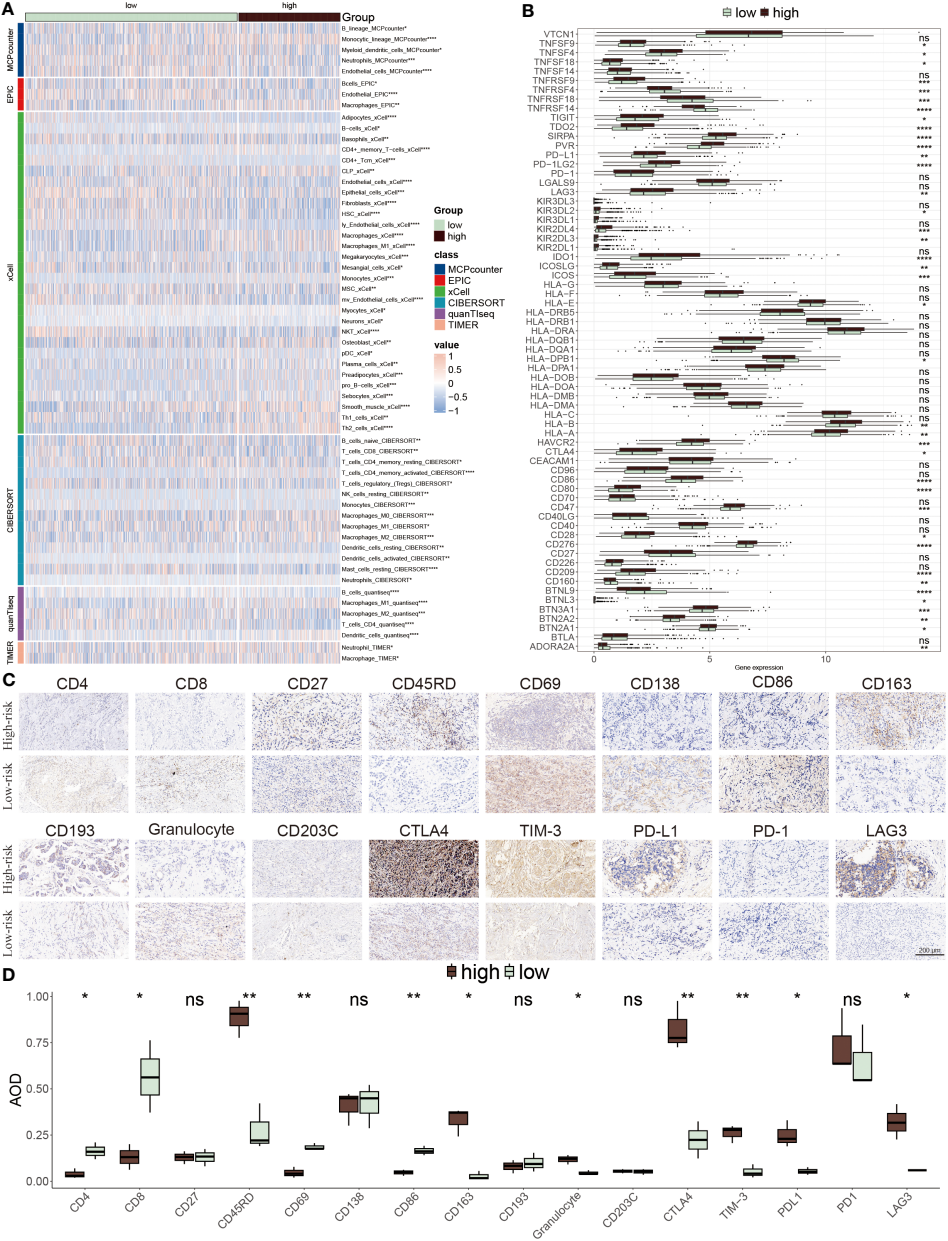
TME evaluation between the ERS-model subgroups. **(A)** The distribution of distinctive infiltrated immune cells between two risk subgroups. **(B)** The differential expression profiles of ICIs between two risk subgroups. *P < 0.05, **P < 0.01, ***P < 0.001, ****P < 0.0001, ns, not significant. **(C)** IHC image of infiltrated immune cells targeting the reproductive makers. **(D)** Statistical result of **(C)**.

We next explored the distinct immune cell infiltration among distinctive groups. In the low-risk patients, extensive immune cells were abundant, for example, native B cells, CD8 T cells, resting CD4 T cells, resting NK cells, and B cells. Conversely, a small number of cells mainly composed of macrophages M0, and M2 were infiltrated in high-risk populations (**Figure 8A**). Interestingly, the expression levels of immune checkpoint inhibitors (ICIs) genes showed that several genes were highly expressed in low-risk groups including ADORA2A, CD27, BTNL9, TNFRSF14, TNFRSF4 and TNFRSF18, while other ICIs notably increased in high-risk group, such as PD-L1, LAG3, CTLA4 and TIM-3 (**Figure 8B**). Representative IHC staining images of the key markers were shown in **Figures 8C, D**.

### Prediction of ICIs therapy based on ERS-model

**Figure 9:**
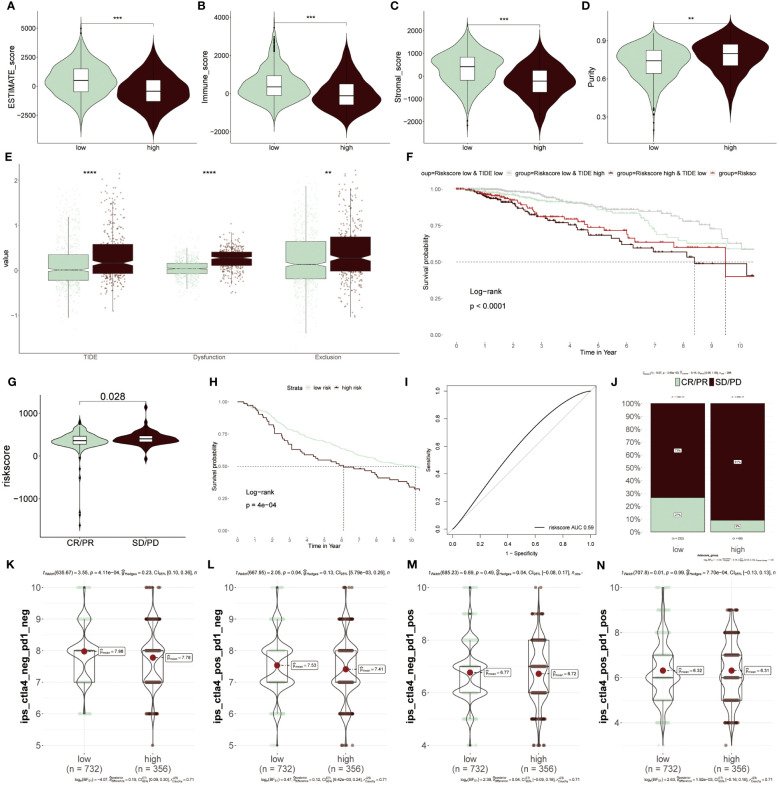
Predicting immunotherapy response using the ERS-Model. **(A–D)** Distinctive scores of Estimate algorithm. **(A)** Estimate scores. **(B)** Immune scores. **(C)** Stromal scores **(D)** Tumor purity. **(E)** Comparison of TIDE, Dysfunction, and Exclusion values between the low- and high-risk groups. **(F)** KM curve displaying survival probability based on the combination of risk score and TIDE. **(G)** Analysis of the relevance between risk score and CR/PR and CD/PD. **(H)** KM survival curve demonstrating the prognostic efficiency of high and low-risk groups after anti-PD-L1 therapy. **(I)** Predictive precision of ERS-model based on AUC values. **(J)** Visualization of the ratios of CR/PR and SD/PD in risk groups using bar charts. **(K–N)** Subtypes of IPS (Immunophenoscore) values among the two risk groups. **P < 0.01, ***P < 0.001, ****P < 0.0001.

To further elucidate which group was more suitable for immunotherapy, the ESTIMATE algorithm was applied. According to results from the ESTIMATE score (**Figure 9A**), immune score (**Figure 9B**) and stromal score (**Figure 9C**), it was found that the low-risk group acquired a higher score relative to the higher group, revealing that immunogenicity was superior in this group. Differently, the tumor purity was higher in high-risk patients, which was related to its inferior survival ability (**Figure 9D**). Moreover, the high-risk group acquired a higher value of TIDE, Dysfunction and Exclusion, of which Dysfunction and Exclusion individually represented tumor immune dysfunction and rejection, thus also confirming that the high-risk group was in the state of immunosuppression (**Figure 9E**). It was detected that patients with low-risk scores and high TIDE possessed longer survival time and better clinical benefits than other combinations, and risk score played a dominantly decisive role (**Figure 9F**). The clinical response diagnosis to immunotherapy targeting PD-L1 (IMvigor210 cohort) was conducted among two groups according to the relevance between risk score and CR (complete response)/PR (partial response) and SD (stable disease)/PD (progressive disease), of which CR/PR possessed a lower risk score as compared to SD/PD, namely equally supporting above results (**Figure 9G**). The result obtained from the KM survival cure confirmed that the low-risk BC patients were superior in prognostic outcomes to other groups after ICIs treatment (**Figure 9H**). Furthermore, the AUC value of the risk score was 0.59, which signified the accuracy of the prediction (**Figure 9I**). Then, the consequence that the low-risk group had a higher proportion of CR/PR relative to the high-risk population, accounting for 27%, was displayed again based on this bar chart (**Figure 9J**). Additionally, IPS was performed to further verify its precision. The low-risk group had greater superiority to anti-PD-1/PD-L1 and anti-CTLA4 therapy in comparison to high-risk patients (**Figures 9K–N**). To integrate the above findings, it was concluded based on ERS-model that these patients of low-risk BC were more suitable for immunotherapy, as well as eight genes related to ERS were able to be recognized as underlying prognostic markers in BC.

### Selection of drug targets and therapeutic candidates for high ERS patients

**Figure 10:**
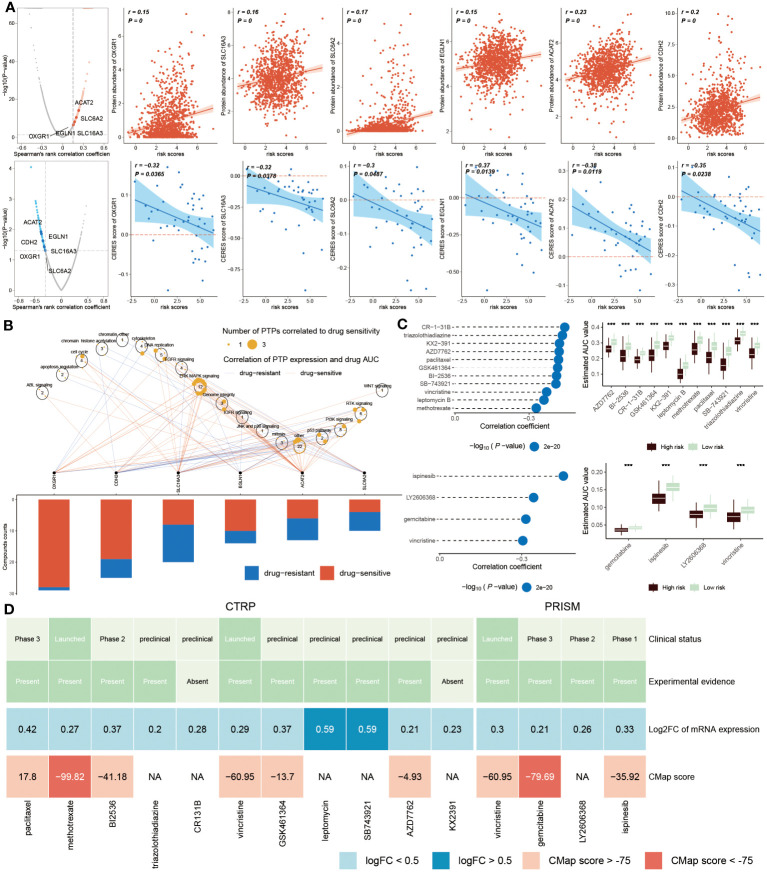
Identification of drug targets and therapeutic agents for high-risk BC. **(A)** Volcano plot showed that the result from Spearman’s correlation analysis, of which r>0.25, P<0.05 represented a remarkably positive relation visualized by red dots. Scatter plots revealed correlations between risk score and protein abundance of drug targets. Similarly, the result of Spearman’s correlation displayed by blue dots represented negative associations (P<0.05, and r<-0.2). The correlations between the risk score and CERES score of drug targets were exhibited by scatter plots. **(B)**. Spearman correlation between mRNA expression of potential targets and drug sensitivity across cancer cell line. **(C)** Spearman’s correlation analysis (left) of 11 compounds obtained from CTRP (top left) and 4 compounds gained from PRISM (bottom left). The boxplot (right) accordingly showed the difference in the estimated AUC value of different compounds within the two groups. **(D)** The diagram displayed the clinical status, experimental evidence, mRNA expression levels and CMap score of eleven agents from CTRP and four agents from PRISM, respectively. ***P < 0.001.

The recognition of therapeutic targets was able to improve the undruggable situation of those proteins due to the lack of active targets. Thus, six druggable therapeutic targets were identified. The result showed that high-risk populations possessed higher gene abundance. CERES score represented the tumor’s reliance on these genes and the degree of reliance was inversely proportional to the CERES score (**Figure 10A**). Consequently, these six genes were capable of being recognized as potential targets for BC treatment, implying that dysfunction of these six genes may be beneficial for BC patients. Meanwhile, these potential drug targets were assessed further according to the proportion of drug-sensitive. Results displayed that six genes were ultimately chosen as the most potential therapeutic targets due to their high drug sensitivity (**Figure 10B**).

We next estimated the drug treatment candidates for high ERS patients from CTRP and PRISM, respectively. CR-1-31B, triazolothiadiazine and other nine compounds derived from CTRP, as well as four compounds, such as ispinesib, LY2606368, gemcitabine and vincristine, obtained from PRISM, were screened out as the candidate drugs (**Figure 10C**). It can be concluded that fifteen compounds possessed lower estimated AUC values in high-risk patients, which implied that this subset may be more suitable for medication treatment. To further select the most potential therapeutic agents from fifteen candidates, a multiple-perspective analysis was conducted. The clinical status and experimental evidence of these compounds were inquired from PubMed. It was found that these candidates had higher fold expression levels, indicating better therapeutic efficiency for BC patients. CMap analysis was carried out to filter those compounds whose expression profiles were opposite to the BC-specific expression feature. The result exhibited that CMap values of methotrexate and gemcitabine were below -75 (**Figure 10D**), suggesting they were equipped with greater potential for treating high ERS BC patients (**Supplementary Table S4**).

## Discussion

In the current study, we discussed the relationship between ERS and BC heterogeneity, of which regulators associated with ERS were acquired from published articles. It was found that a variety of ERS genes were abnormally expressed in normal and BC samples, indicating the dislocation of ERS was closely related to the progression of BC. At the same time, the analysis based on the levels of transcriptome and single-cell was conducted, discovering that ERS score was high in normal populations relative to BC patients, and its levels were proportional to T cells, and B cells et al., implying the inferior prognosis of BC patients. Since the single-cell analysis was beneficial to better understand tumors, we then explored the connection between ERS and BC at the single-cell level and found that ERS levels exhibited a positive relation with immune cell infiltration. Moreover, the normal population had a higher ERS score, inferring that the connection between high ERS score and immune activation was an underlying mechanism to improve BC patients’ outcomes. Previous research mentioned that tumor unfolded protein response exerted a tumor-promoting effect via attenuating the activity of CD8^+^ T cells (43). While the activation of immune cells, especially T cells, combined with targeted ERS urgently demanded to be considered as a novel strategy for BC patients, based on the efficiency of the drugs targeting ERS (44). Additionally, the relationships of immune cells in BC patients were explored, indicating a complex interaction in the progression of BC. Moreover, we explored the interaction numbers and strength of immune cells in BC groups, revealing that stronger interaction existed between macrophages, pDC cells and monocytes. At the same time, the result from outgoing and incoming interaction strength also demonstrated macrophages and pDC cells showed stronger interaction in BC samples relative to normal groups. Furthermore, we found that the interaction of massive ligand-receptors mainly occurred in BC patients with macrophage interaction, while T cells and B cells were more prominent in normal individuals. In conclusion, it indicated that the complicated interaction among these immune cells, especially macrophages, exerted significant roles in BC development but the mechanism demanded to be researched further.

Highlighting the significant roles of ERS in BC prognosis, we developed a prognostic model using eight ERS-related genes. This model was evaluated in one training set and three validation sets, and it was compared with five existing models to demonstrate its efficacy. Our results demonstrated that ERS prognostic model possessed a robust, independent and reliable performance. In the ERS-model, BC patients were successfully divided into two subgroups, of which low-risk BC patients were characterized by better survival status, longer overall survival, and fewer deaths. Since then, ERS-nomogram was established to forecast the survival probability of BC patients at 1, 3 and 5 years, which had an optimum predictive ability as compared to other clinicopathological characteristics based on the AUC values. Among them, it was worth noting that eight ERS genes displayed distinctive expression levels among two risk groups. SRPRB, DAB2IP, HSPA8 and GFAP were up-regulated in high-risk groups, while KCNJ1, ERP27, SERPINA3 and AVP were mainly concentrated in low-risk patients.

SRPRB, also known as APMCF1, exerted a critical role in the proliferation and progression of cells. Another study offered evidence that SRPRB is highly expressed in apoptotic MCF-7 cells (45). Additional research found that it was recognized as a clinical prognostic molecule in multiple myeloma (46). Furthermore, in pancreatic ductal adenocarcinoma, SRPRB was regulated by SERP1, a prognostic marker, acting on its development (47). In our study, it presented a higher level in high-risk BC patients, which also hinted at the inferior outcome in this group and was listed as a potential target for BC treatment. DAB2IP belonged to the Ras GTPase-activating protein family and played an anti-tumor role in multiple cancers, such as esophageal squamous cell carcinoma (48), and triple-negative breast cancer (49). Additionally, in TBNC, it was shown that the expression of DABI2P was able to alleviate chemoresistance (49), which means that it was a promising market to improve the prognosis of BC patients. HSPA8, a member of the heat shock protein family, participated in protein folding. In the development of cancer, increasing studies indicated that it served as a promoter in the progression of tumors. For example, HSPA8 was triggered to advance liver cancer development by boosting HBV replication and inhibiting ferroptosis (50). Likewise, in acute myeloid leukemia, it also was reported that HSPA8 had a negative relation with tumor suppressors (51). Another study showed that HSPA8 had a higher expression level in high-risk TNBC patients (52), which was in line with our study, manifesting that its abnormal expression was linked to the inferior prognosis of BC patients. Similarly, GFAP had been recommended as a prognostic marker in distinctive cancers, such as malignant astrocytoma (53). Similarly, a previous study declared that GFAP was associated with poor outcomes in BC patients (54). KCNJ1 participated in potassium balance, and its mutation was able to trigger Bartter syndrome (55). In clear cell renal cell carcinoma, the results indicated that this gene was highly expressed in normal tissue samples and produced an inhibitory role in ccRCC progression (56), while there was a lack of reports about its role in BC. ERp27 takes part in ER stress response by cooperating with ERp57 (57), which had been considered a novel prognostic marker among various cancers including pancreatic ductal adenocarcinoma (58) and breast cancer (11). SERPINA3 belongs to the SERPIN family and acts as a protease inhibitor to maintain cellular homeostasis (59). Differently, numerous research revealed that SERPINA3 functioned as a promoter in cancer progression. In GMB and colon cancer, the silencing of this gene was able to suppress these cells’ growth and migration (60), unless it increased the incidence of GMB in older patients (61). Equally, related research claimed that the high expression of SERPINA3 was negatively relevant to the GMB prognosis (62). In TNBC, the high expression levels of SERPINA3 resulted in TNBC cell proliferation (63). Finally, AVP, also known as an antidiuretic hormone, secreted by the hypothalamus, plays a significant role in enhancing the permeability of the collecting tube to water to promote water absorption to maintain homeostasis. Hyponatremia induced by AVP increased treatment risk and brought adverse reactions including pain et al. in cancer patients (64). However, the function of AVP remains unclear in BC. Altogether, except for the AVP gene, other genes had been reported as candidates for various cancers treatment, including breast cancer. Accordingly, the upregulation or downregulation of eight genes among different groups was tightly relevant to the prognosis of BC patients and their roles were worth in-depth investigation in BC.

Since then, the distinction of gene alteration between the two risk groups was described. It was concluded that low-risk BC patients were featured by lower TMB and low-mutated genes, while high-risk BC patients possessed more TMB and more mutated genes, such as TP53. Massive research had reported that its mutation promoted the progression of BC, resulting in an inferior prognosis (65). On the other hand, CNV analysis showed that the amplification of 3q26.32, 5p15.33 and 8q24.21, and the deletion of 5q21.3, 8p23.2 and 9p21.3 were primarily founded in high-risk patients. Kusakabe et al. found that the fusion of HPV18 and chromosome 8q24.21 was capable of enhancing the expression level of the MYC gene (66), suggesting that it further promoted the development of tumors because of the role of MYC in accelerating tumor progression. But there was no study about its function in BC. The deletion of 8p23.2 had been reported that could induce advanced liver cancer (67). Lu et al. described that its deletion may enhance the metastasis of HCC (68). Although these characteristics highlighted the poor prognosis of BC patients, however, it also laid the foundation for the response to ICI therapy in low-risk patients. Meanwhile, GSEA analysis revealed that immune-related signaling pathways, such as antigen processing and presentation, protein processing in the endoplasmic reticulum and negative regulation of natural killer cell-mediated immunity, were inhibited in high-risk patients, which intimated its potential for receiving immunotherapy. Then, we evaluated the immune feature between the two risk groups, the results showed that low-risk patients possessed a high proportion of immune cell infiltration, especially T and B cells. In contrast, M2 macrophage cells were mainly infiltrated in high-risk groups. More intriguingly, in low-risk patients, these up-regulated genes were more related to targeted therapy, such as TNFRSF14, TNFRSF4, TNFRSF18 and CD27. Related studies claimed that TNFRSF14, TNFRSF4 and TNFRSF18 mainly participated in the proliferation and survival of CD8^+^ and CD4^+^ T cells, so exerted significant roles in immunoregulation (69). Similarly, CD27 also has been recognized as a therapeutic target, because of offering a co-stimulatory signal to T cells (70). These clues highlighted the advantages of this subset in immunotherapy and good prognosis. By comparison, those up-regulated immunity genes stood out in the connection with macrophages. For example, CD68 and CD80 are markers of macrophages, BTN2A2 express on the peritoneum of macrophages and inhibits T cells activity (71), as well as CD47 joins in macrophage phagocytosis (72). Terminally, responsiveness to ICIs therapy among distinctive subgroups was estimated, proven by facts, low-risk groups were superior in immunotherapy, based on their high immunogenicity.

Different from the low-risk subgroup, high-risk patients were more inclined to drug therapy. The truth was that those proteins that were related to ERS and in possession of the underlying ability of BC treatment remained undruggable due to the lack of the binding site of small molecules. Therefore, this study identified therapeutic targets, and ultimately six drug targets were chosen. Moreover, two therapeutic agents, methotrexate and gemcitabine, were also identified. Methotrexate is an anti-cancer medicine and has been widely used, including in ovarian cancer (73). Kapke et al. reported that high-dose methotrexate could prolong the survival time for BC patients (73). Shakeran et al. performed a study on drug combination, finding that combining methotrexate and STAT3 siRNA was efficient in improving the therapeutic efficiency of BC (74). Another agent gemcitabine was also a therapeutic drug reported widely in multiple cancers, such as advanced biliary tract cancer (75), and pancreatic ductal adenocarcinoma (76). In breast cancer, Yardley DA claimed that whether used in combination with taxanes or alone, gemcitabine was efficient for BC therapy (77). Above all, these results enhanced the persuasiveness of these two agents used for the treatment of high-risk BC patients.

## Conclusion

Our investigation has elucidated a crucial association between ERS and the progression of breast cancer, analyzed through both bulk and single-cell methodologies. Central to our study was the development of an ERS-model predicated on eight genes significantly correlated with ERS, which has demonstrated high accuracy in forecasting the clinical outcomes for BC patients.

Further, by categorizing BC patients into two distinct risk subgroups, our analysis shed light on their differential functional enrichment, immune cell composition, and varying responses to ICIs and chemotherapeutic agents. Notably, our work led to the identification of key therapeutic targets and drugs, markedly enhancing the translational value of our findings. Specifically, we uncovered that ICIs treatment may be preferentially beneficial for patients classified within the low-risk category, whereas chemotherapeutic approaches showed augmented efficacy in managing high-risk BC patients. This strategic patient stratification culminated in pinpointing six promising drug targets and two particularly potent therapeutic agents, offering new hope and potential treatment pathways for individuals facing higher risk factors.

Ultimately, our study contributes significantly to the nuanced understanding of breast cancer dynamics and lays the groundwork for more personalized, risk-adjusted therapeutic interventions. By bridging critical gaps in our knowledge of ERS’s role in BC and leveraging this understanding to inform treatment selection, we are poised to enhance patient care and optimize treatment outcomes in breast cancer.

## Data availability statement

The datasets presented in this study can be found in online repositories. The names of the repository/repositories and accession number(s) can be found in the article/**Supplementary Material**.

## Ethics statement

The studies involving humans were approved by the Ethics Committee of Guizhou Provincial People’s Hospital. The studies were conducted in accordance with the local legislation and institutional requirements. The participants provided their written informed consent to participate in this study.

## Author contributions

BY: Data curation, Formal analysis, Software, Visualization, Writing – original draft. SW: Data curation, Investigation, Methodology, Software, Writing – original draft. YY: Formal analysis, Investigation, Methodology, Software, Validation, Writing – original draft. XL: Investigation, Methodology, Validation, Writing – original draft. FY: Conceptualization, Supervision, Writing – review & editing. TW: Conceptualization, Formal analysis, Funding acquisition, Investigation, Methodology, Supervision, Validation, Writing – review & editing.
